# Blastic plasmacytoid dendritic cell neoplasm presenting as a scalp hematoma

**DOI:** 10.1016/j.jdcr.2026.01.018

**Published:** 2026-01-22

**Authors:** Eduardo A. Michelen-Gómez, Karina Cancel-Artau, Cristina P. Gerena-Maldonado, Iancarlos Jiménez-Sacarello, María Rivera-Rolón, Julio E. Sánchez-Pont, Rafael F. Martín-García

**Affiliations:** a Department of Dermatology, University of Puerto Rico School of Medicine, San Juan, Puerto Rico; b School of Medicine, University of Puerto Rico Medical Sciences Campus, San Juan, Puerto Rico; c Department of Pathology, University of Puerto Rico School of Medicine, San Juan, Puerto Rico

**Keywords:** blastic plasmacytoid dendritic cell neoplasm, tagraxofusp, targeted therapy

## Case description

A 69-year-old male presented to the dermatology clinic with a 5-month history of a progressively enlarging, tender scalp lesion that developed after a minor scalp trauma. Past medical history included hypertension, polycythemia vera, dyslipidemia, and coronary artery disease. The patient reported recent-onset lesions in his chest in addition to significant weight loss of 60 pounds over the past 6 months, accompanied by fatigue and sporadic night fevers. Previous imaging (CT/MRI) ordered by his hematologist were interpreted as hematoma.

**Fig 1:**
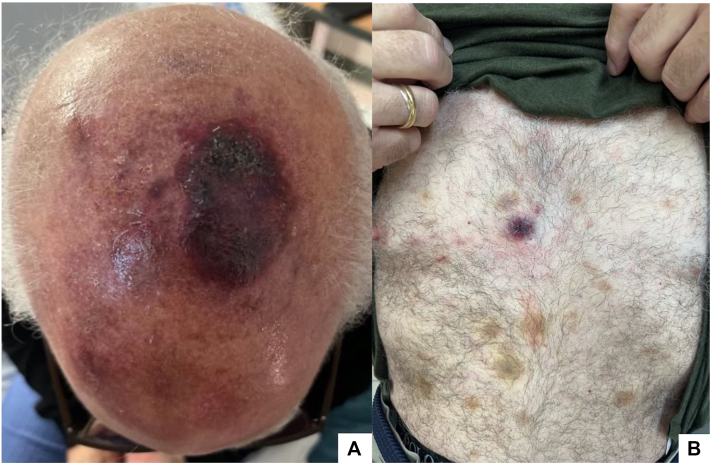
Clinical presentation: **A,** Indurated hemorrhagic plaque localized to the right parietal scalp surrounded by *smaller* similar lesions. **B,** Violaceous indurated plaque and ecchymotic patches on chest and abdomen.

Upon physical examination, a 7.0 cm hemorrhagic plaque was evident on the left parietal scalp (Fig 1) surrounded by purpuric patches and papules. Ecchymotic macules, patches, and plaques were present in the chest and abdomen. No palpable lymphadenopathy or hepatosplenomegaly was identified.

**Fig 2:**
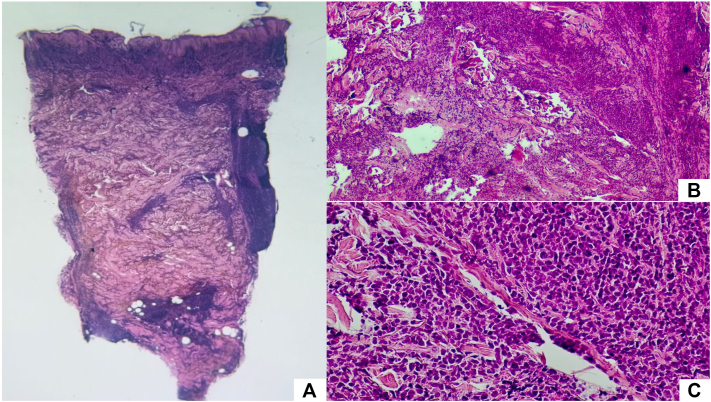
**A,** Superficial and deep dense nodular infiltrate of mononuclear cells (H&E, 2×). **B,** Involvement of adnexal structures and vasculature (H&E, 10×). **C,** Immature blastic cells with nuclear irregularities, prominent nucleoli, chromatin, and amphophilic cytoplasm (H&E, 20×).

**Fig 3:**
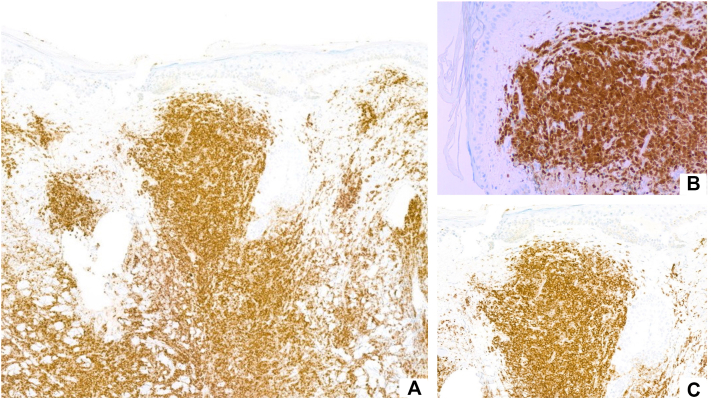
Immunohistochemical staining: **A,** CD123 positivity (10×). **B,** TCL1 positivity (20×). **C,** CD123 positivity (20×).

Histopathologic examination of the scalp plaque biopsy revealed a dense dermal infiltration of medium-sized blastic cells with irregular nuclear contours, open chromatin, and scant cytoplasm (Fig 2). Immunohistochemical staining (Fig 3) was positive for CD123, TCL1, CD4, and CD56, confirming the diagnosis of blastic plasmacytoid dendritic cell neoplasm (BPDCN).

18F-FDG PET/CT demonstrated hypermetabolic cutaneous lesions in the scalp, right anterior chest wall, and left proximal thigh, as well as a focal hypermetabolic intramedullary lesion in the right mid-tibia and diffuse increased bone marrow FDG uptake, supporting systemic involvement.

**Fig 4:**
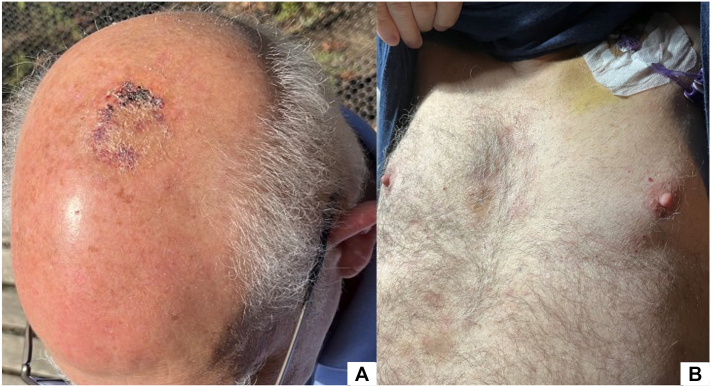
Clinical improvement after 2 cycles of treatment. Marked regression of hemorrhagic plaques in scalp **(A)** and chest **(B)**.

The patient was referred to a tertiary center and enrolled in a trial, receiving combination therapy with venetoclax, azacitidine, and tagraxofusp. After completing 2 cycles, the patient demonstrated significant regression of cutaneous lesions (Fig 4) and alleviation of systemic symptoms. The patient underwent a successful allogeneic hematopoietic cell transplantation (allo-HCT) and has been free of disease for 8 months.

**Question: What is the mechanism of action of tagraxofusp in BPDCN?**

**A.** Blocks the IL-3 receptor β-chain (CD131), inhibiting Ras/MAPK signaling.
**B.** Recombinant fusion protein of human IL-3 with a diphtheria toxin that binds CD123, enters the cell, and inhibits protein synthesis, inducing cell death.
**C.** Antibody–drug conjugate targeting CD123 that delivers a cytotoxic agent.
**D.** Small-molecule tyrosine-kinase inhibitor against CD123.
**E.** Increases CD123 expression to promote anti-tumor immunity.

## Discussion

BPDCN is a rare and aggressive hematologic malignancy that typically presents with cutaneous lesions.^1^ Histopathologically, co-expression of CD123 and CD56 is particularly suggestive of this condition.^2^ The treatment landscape for BPDCN has evolved substantially. Historically, multiagent chemotherapy regimens led to high initial response rates but frequent relapses, with median overall survival ranging from 8 to 14 months.^3^ The advent of targeted therapies including tagraxofusp has improved patient outcomes. Answer choice B correctly describes the mechanism of action of tagraxofusp. This recombinant fusion protein delivers diphtheria toxin via CD123, which is highly expressed on BPDCN cells, inhibiting protein synthesis and inducing selective cytotoxicity.^3^ A prospective study reported a median overall survival of 15.8 months in treatment-naive patients.^4^ Among those who achieved remission and proceeded to allo-HCT, survival improved significantly, with a median overall survival of 38.4 months and a 2-year survival rate of 66%.^4^ The multimodal regimen our patient received aims to target BPDCN cells through multiple pathways: CD123-directed cytotoxicity with tagraxofusp, apoptosis induction via BCL-2 inhibition with venetoclax, and enhanced cytotoxicity with azacitidine. Our patient responded excellently to this multimodal chemotherapy followed by allo-HCT, underscoring the potential role of this therapy in patients with BPDCN.

## Conflicts of interest

None disclosed.
